# Reared adults of *Potamanthellus unicutibius* Nguyen & Bae, 2003 from China (Ephemeroptera, Neoephemeridae)

**DOI:** 10.3897/BDJ.14.e211811

**Published:** 2026-09-29

**Authors:** Dewen Gong, Changfa Zhou

**Affiliations:** 1 Nanjing Normal University, Nanjing, China Nanjing Normal University Nanjing China

**Keywords:** evolution, imago, mayfly, Oriental realm, taxonomy

## Abstract

**Background:**

The nymphs of *Potamanthellus unicutibius* Nguyen & Bae, 2003 possess filtering mouthparts and modified fore-legs. However, no adults are known.

**New information:**

In this study, both nymphal and imaginal stages of this species were collected and reared from China. Morphological examination reveals that these specimens differ markedly from all other members of *Potamanthellus* Lestage, 1931. In addition to the filtering nymphal morphology, the males of this species are characterised by more distinctly developed penes, longer tibiae and cerci and smaller compound eyes compared to those of *Potamanthellus*. Significantly, its distinct penes not only validates its independent species status, but also reveals the evolutionary process of the reduction of neoephemerid male genitalia and provides the original clue of the vestigial penes in several other lineages of the order Ephemeroptera as well.

## Introduction

The species *Potamanthellus unicutibius* was originally described by Nguyen and Bae (2003), based on nymphs collected from Vietnam. Since then, no further report on this species has been published.

Unlike any other member of the family Neoephemeridae, nymphs of *P. unicutibius* possess filtering fore-legs, with both fore-tibiae and fore-tarsi bearing long, brush-like setae — a morphological trait typically observed in Isonychiidae or Oligoneuriidae (Qiang and Zhou 2023, Chen et al. 2025). Their mouthparts also exhibit a filtering configuration, as both maxillary and labial palpi are elongated and equipped with long hair-like setae. By contrast, all other genera and species within the same family are characterised by robust, chewing or cutting mouthparts and their bodies generally lack elongate setae (Bae and McCafferty 1998, Ma and Zhou 2021, Li et al. 2026). In their original description, Nguyen and Bae (2003) noted that these nymphs likely warranted the erection of a new genus, but emphasised that adult specimens were necessary for a definitive taxonomic decision.

Currently, the family Neoephemeridae comprises five extant genera and 17 described species (Bae and McCafferty 1998, Ma and Zhou 2021, Li et al. 2026). Based on characters of the male imaginal genitalia, nymphal gills and caudal filaments, these genera can be divided into two distinct morphological groups: the *Neoephemera*-like group, characterised by well-developed male forceps, two operculate gills II that are connected or jointed medially and nymphal caudal filaments bearing dense spines; and the *Potamanthellus*-like group, which possesses vestigial male forceps, free gills II and nymphal tails with dense setae and spines. The former group includes four genera: *Leucorhoenanthus* Lestage, 1931; *Neoephemera* Mcdunnough, 1925; *Ochernova* Bae and McCafferty, 1998; and *Pulchephemera* Ma and Zhou, 2021 (Lestage 1931, Mcdunnough 1925, Bae and McCafferty 1998, Ma and Zhou 2021Lestage 1931, ), whereas the latter currently contains only a single genus, *Potamanthellus* Lestage, 1931 (Kluge 2004, Ma and Zhou 2021). Further discoveries within this second clade would not only enhance our understanding of its species diversity, but also provide valuable insights into its evolutionary trajectories.

In spring 2026, we conducted a collecting expedition in Yunnan Province, south-western China. During this survey, dozens of *P. unicutibius* nymphs were collected. Through careful indoor rearing, more than ten adults were successfully obtained. These newly-acquired materials provide a valuable opportunity to describe the previously unknown adult stage and to reassess the taxonomic and phylogenetic status of this enigmatic species.

## Materials and methods

### Specimen collection and storage

Nymphs were collected using hand nets and adults were obtained both by rearing mature nymphs under laboratory conditions and by light trapping. All specimens were preserved in 80% or higher concentration ethanol and are currently deposited in the Mayfly Collection, College of Life Sciences, Nanjing Normal University (NNU), China.

For rearing, mature nymphs were placed in trays containing natural water, along with small stones, twigs and leaves. Each tray was covered with a small tent constructed from mosquito netting. Throughout the rearing period, a small aerator was used to continuously supply air to the water. Emerging adults were collected from inside the tent as they emerged.

### Illustrations

Morphological observations were conducted using a combination of imaging techniques. Whole-body images of both nymphs and adults were captured with a Sony A7RIV interchangeable-lens digital camera, equipped with a 90 mm macro lens. For intermediate-sized structures, such as male genitalia and hind-wings, a Sony A7RIV camera, fitted with a Laowa FF 25 mm F2.8 Ultra Macro lens, was used. Egg morphology was examined using a Thermo Fisher Scientific Apreo 2S scanning electron microscope (SEM).

Eggs for SEM analysis were dissected from female imagoes. Together with the hind-legs of female subimagos, they were processed following a standard dehydration protocol: sequential immersion in an acetone gradient (70%, 80%, 90%, 98% and 100%, 10–20 min per step), followed by treatment with 1–1.5 ml of hexamethyldisilazane (HMDS) for 30 min. The specimens were then dried overnight in a vacuum desiccator and subsequently sputter-coated with a gold layer prior to imaging.

## Taxon treatments

### Potamanthellus unicutibius

Nguyen & Bae, 2003

749A1C66-C85D-5805-B5E1-CA3C8B9E97E9

Potamanthellus unicutibius Nguyen & Bae, 2003 - Nguyen and Bae (2003): 233. Types: nymph, from Vietnam.
Potamanthellus unicutibius : Ma and Zhou (2021): 2 (mentioned); Garces and Sartori (2022): 49 (mentioned).

#### Materials

- **Type status:** Other material. **Occurrence:** recordedBy: De–Wen Gong, Xu–Hong–Yi Zheng; individualCount: 1; sex: female; lifeStage: imgao; occurrenceID: 81807E76-7F97-50AD-B8A3-D4B2DD7633F0; **Taxon:** scientificName: *Potamanthellus unicutibius*; **Location:** country: China; stateProvince: Yunnan; county: Yinjiang; locality: Nabang town, Yinjiang county, Yunnan Province, China; verbatimElevation: 1106 m; verbatimCoordinates: 24°37.39'N 97°35.02'E; decimalLatitude: 24.623242; decimalLongitude: 97.583675; georeferenceProtocol: label; **Event:** eventDate: 18/19-04-2026; **Record Level:** language: en; collectionCode: Insects; basisOfRecord: PreservedSpecimen
- **Type status:** Other material. **Occurrence:** recordedBy: De–Wen Gong, Xu–Hong–Yi Zheng; individualCount: 20; lifeStage: nymph; occurrenceID: 40F6470E-8283-57F6-87AC-A7DF34A40F12; **Taxon:** scientificName: *Potamanthellus unicutibius*; **Location:** country: China; stateProvince: Yunnan; county: Yinjiang; locality: Nabang town, Yinjiang county, Yunnan Province, China; verbatimElevation: 1106 m; verbatimCoordinates: 24°37.39'N 97°35.02'E; decimalLatitude: 24.623242; decimalLongitude: 97.583675; georeferenceProtocol: label; **Event:** eventDate: 18/19-04-2026; **Record Level:** language: en; collectionCode: Insects; basisOfRecord: PreservedSpecimen
- **Type status:** Other material. **Occurrence:** recordedBy: De–Wen Gong, Di Wang; individualCount: 1; sex: female; lifeStage: subimago; occurrenceID: 31F914E4-427F-5D22-9AB2-6492C2AA0CB3; **Taxon:** scientificName: *Potamanthellus unicutibius*; **Location:** country: China; stateProvince: Yunnan; county: Yinjiang; locality: Nabang town, Yinjiang county, Yunnan Province, China; verbatimElevation: 1106 m; verbatimCoordinates: 24°37.39'N 97°35.02'E; decimalLatitude: 24.623242; decimalLongitude: 97.583675; georeferenceProtocol: label; **Event:** eventDate: 8/10-05-2026; **Record Level:** language: en; collectionCode: Insects; basisOfRecord: PreservedSpecimen
- **Type status:** Other material. **Occurrence:** recordedBy: De–Wen Gong, Di Wang; individualCount: 1; sex: male; lifeStage: subimago; occurrenceID: E97D1E5C-769B-5B26-BE08-1AA72F1821A7; **Taxon:** scientificName: *Potamanthellus unicutibius*; **Location:** country: China; stateProvince: Yunnan; county: Yinjiang; locality: Nabang town, Yinjiang county, Yunnan Province, China; verbatimElevation: 1106 m; verbatimCoordinates: 24°37.39'N 97°35.02'E; decimalLatitude: 24.623242; decimalLongitude: 97.583675; georeferenceProtocol: label; **Event:** eventDate: 8/10-05-2026; **Record Level:** language: en; collectionCode: Insects; basisOfRecord: PreservedSpecimen
- **Type status:** Other material. **Occurrence:** recordedBy: De–Wen Gong, Di Wang; individualCount: 5; sex: male; lifeStage: imago; occurrenceID: A96BB679-F6D4-55FD-968C-E24A2EAE7BA5; **Taxon:** scientificName: *Potamanthellus unicutibius*; **Location:** country: China; stateProvince: Yunnan; county: Yinjiang; locality: Nabang town, Yinjiang county, Yunnan Province, China; verbatimElevation: 1106 m; verbatimCoordinates: 24°37.39'N 97°35.02'E; decimalLatitude: 24.623242; decimalLongitude: 97.583675; georeferenceProtocol: label; **Event:** eventDate: 8/10-05-2026; **Record Level:** language: en; collectionCode: Insects; basisOfRecord: PreservedSpecimen
- **Type status:** Other material. **Occurrence:** recordedBy: De–Wen Gong, Di Wang; individualCount: 6; sex: female; lifeStage: imago; occurrenceID: 5C0FF554-F6EF-51F3-BA46-0534F832C924; **Taxon:** scientificName: *Potamanthellus unicutibius*; **Location:** country: China; stateProvince: Yunnan; county: Yinjiang; locality: Nabang town, Yinjiang county, Yunnan Province, China; verbatimElevation: 1106 m; verbatimCoordinates: 24°37.39'N 97°35.02'E; decimalLatitude: 24.623242; decimalLongitude: 97.583675; georeferenceProtocol: label; **Event:** eventDate: 8/10-05-2026; **Record Level:** language: en; collectionCode: Insects; basisOfRecord: PreservedSpecimen
- **Type status:** Other material. **Occurrence:** recordedBy: De–Wen Gong, Di Wang; individualCount: 15; lifeStage: nymph; occurrenceID: D3B76374-BDD2-55C0-9828-078CE2FB9A37; **Taxon:** scientificName: *Potamanthellus unicutibius*; **Location:** country: China; stateProvince: Yunnan; county: Yinjiang; locality: Nabang town, Yinjiang county, Yunnan Province, China; verbatimElevation: 1106 m; verbatimCoordinates: 24°37.39'N 97°35.02'E; decimalLatitude: 24.623242; decimalLongitude: 97.583675; georeferenceProtocol: label; **Event:** eventDate: 8/10-05-2026; **Record Level:** language: en; collectionCode: Insects; basisOfRecord: PreservedSpecimen

#### Description

**Figure 1.**
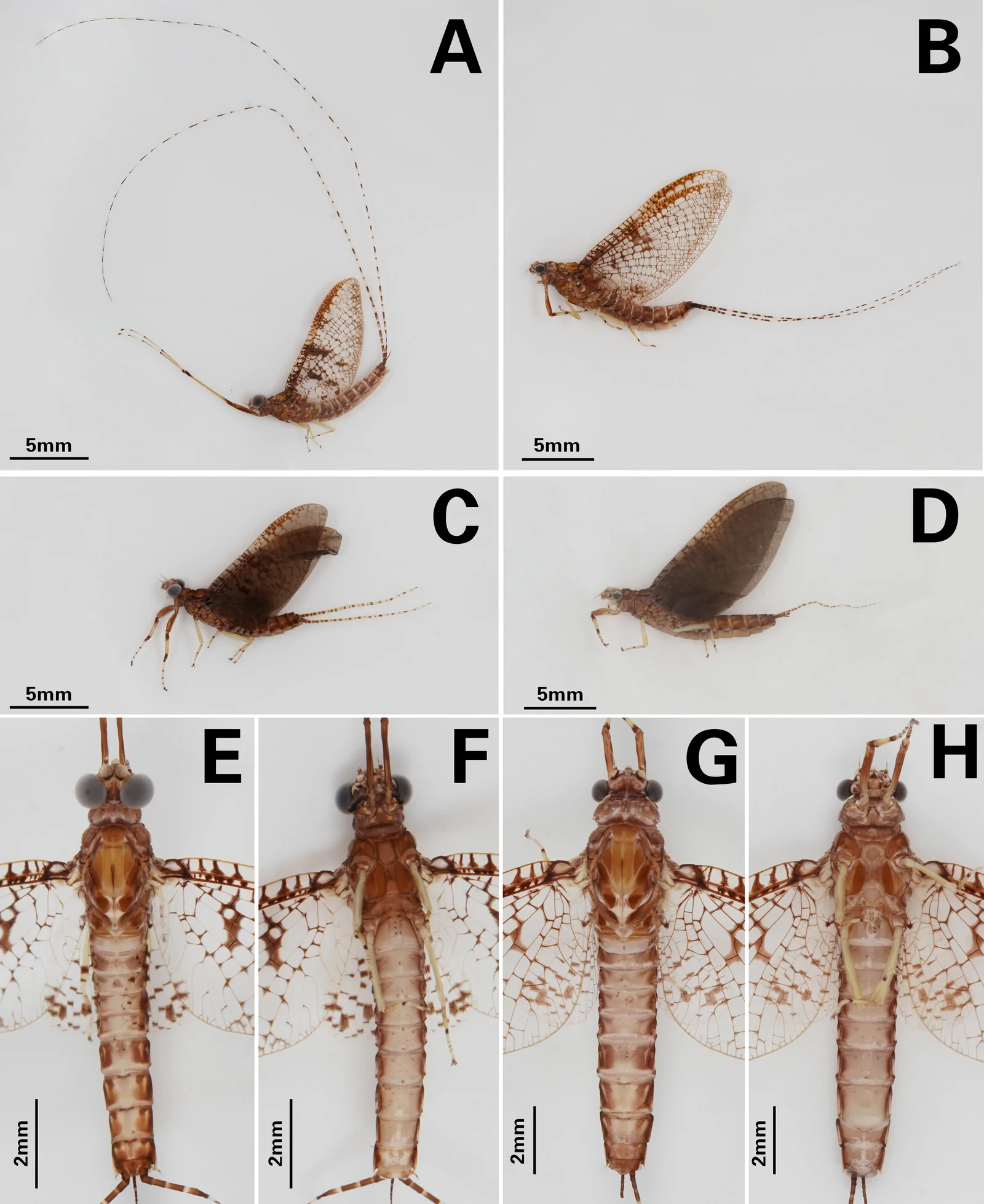
Imaginal habitus of *Potamanthellus unicutibius* Nguyen & Bae, 2003 **A** Male imago; **B** Female imago; **C** Male subimago; **D** Female subimago; **E** Male imago (dorsal view); **F** Male imago (ventral view); **G** Female imago (dorsal view); **H** Female imago (ventral view).

**Figure 2.**
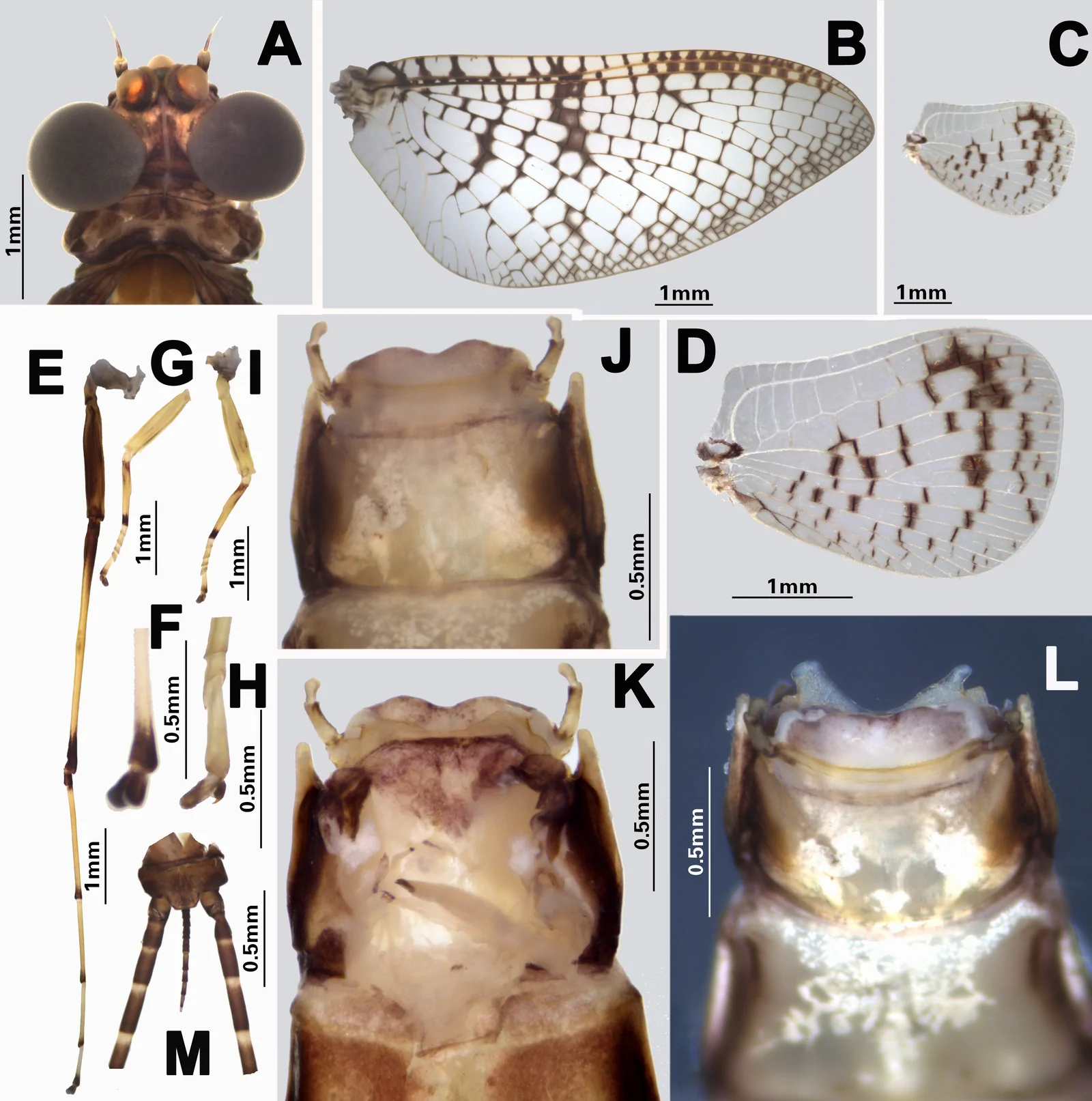
Characters of male imago of *Potamanthellus unicutibius* Nguyen & Bae, 2003 **A** Head (dorsal view); **B** Fore-wing; **C** Hind-wing; **D** Hind-wing enlarged; **E** Fore-leg; **F** Fore-claw; **G** Mid-leg; **H** Mid-claw; **I** Hind-leg; **J** Genitalia (ventral view); **K** Genitalia (dorsal view); **L** Genitalia (apicodorsal view, showing penes).

Male imago (in alcohol): body length 8.0–10.0 mm, fore-legs 11.0–12.0 mm, fore-wings 9.0–9.5 mm, hind-wings 3.0–3.2 mm, cerci 40.0–42.0 mm, terminal filament 1.0–1.1 mm; body reddish-brown (Fig. 1A and E–F). Antennae brown, but apical half of scape and flagella whitish; antennae narrowed from base to apex (Fig. 2A); vertex white to grey, but with a brown stripe between two compound eyes (Fig. 2A). Ocelli cylindrical, with chocolate basal rings; lateral ocelli distinctly larger than median one (Fig. 2A). Compound eyes divided indistinctly into two parts, shortest distance between two eyes about 0.3x diameter of one eye in dorsal view, just slightly wider than median ocellus, but narrower than lateral ocellus (Fig. 2A). Posterior margin of occiput almost straight (Fig. 2A). Pronotum brown, posterior margin concave (Fig. 1E and Fig. 2A). Mesonotum with four pairs of deep brown dots laterally and one brown median pit (or called medial ocellus, Fig. 1E). Prosternum triangular, mesothoracic furcasternum with almost straight inner margins (Fig. 1F). Fore-wings transparent, but longitudinal veins brown, all crossveins pigmented reddish-brown and surrounding clouds (Fig. 1A and E–F), especially those near margins; MA forked distally to middle point; MP forked at base (Fig. 2B and Fig. 8B). Hind-wings also with various reddish to chocolate dots and markings, length ca. 1.5x width (Fig. 2C); costal projection blunt; R1 straight and zigzag; MA single, MP forked at middle point almost symmetrically (Fig. 2D and Fig. 8C). Femora of fore-legs brown to chocolate, tibiae with brown base and apex, segment I of fore-tarsi dark brown, segments II–V with brown apex, other parts yellow (Fig. 2E). Length ratio of fore-femora: tibiae: tarsi = 1.0: 2.3: 2.7, length ratio of five segments of fore-tarsi = 1.0: 6.3: 4.7: 3.7: 2.3 (Fig. 2E); two claws of fore-legs black and blunt (Fig. 2F). Tibiae of mid-legs with brown base and dark apex, tarsi with dark apex too; length ratio of mid-femora: tibiae: tarsi = 1.0: 0.9: 0.6, length ratio of four movable segments of mid-tarsi = 1.0: 1.0: 0.5: 2.5 (Fig. 2G); claws black, one blunt one acute (Fig. 2H). Hind-legs similar to mid-legs, length ratio of mid-femora: tibiae: tarsi = 1.0: 0.9: 0.6, length ratio of four movable segments of mid-tarsi = 1.0: 0.8: 0.5: 2.0 (Fig. 2I). Mid- and hind-tibiae with patellar-tibial fusion suture (Fig. 2G and I). Terga of abdomen reddish-brown, washed with indistinct pale stripes (Fig. 1E); sterna pale, but with brown lateral margins (Fig. 1F). Tergum IX with distinct posterolateral projections (Fig. 2J–K and Fig. 8D–E). Genitalia: subgenital plate with almost straight posterior margin, width about 3.5x length (Fig. 2J); forceps slightly longer than length of subgenital plate, length ratio of segment I: II = 1.0: 0.6; segment I narrowed slightly from base to apex, apex dark; segment II with slightly expanded apex (Fig. 2J–K). Penes membranous, ventral surface grey, slightly extended out of subgenital plate; gonopore at posterolateral corner of penis (Fig. 2L); a dorsal plate between penes and subanal plate, posterolateral corner of this plate slightly extended outwards forming an acute apex (Fig. 2K). Cerci with two kinds of segments: apical half chocolate segment and basal dark ring segment, two kinds of them arranged alternately (Fig. 1A and E–F). Terminal filament black, with 8–10 segments, distinctly thinner than cerci (Fig. 2M).

**Figure 3.**
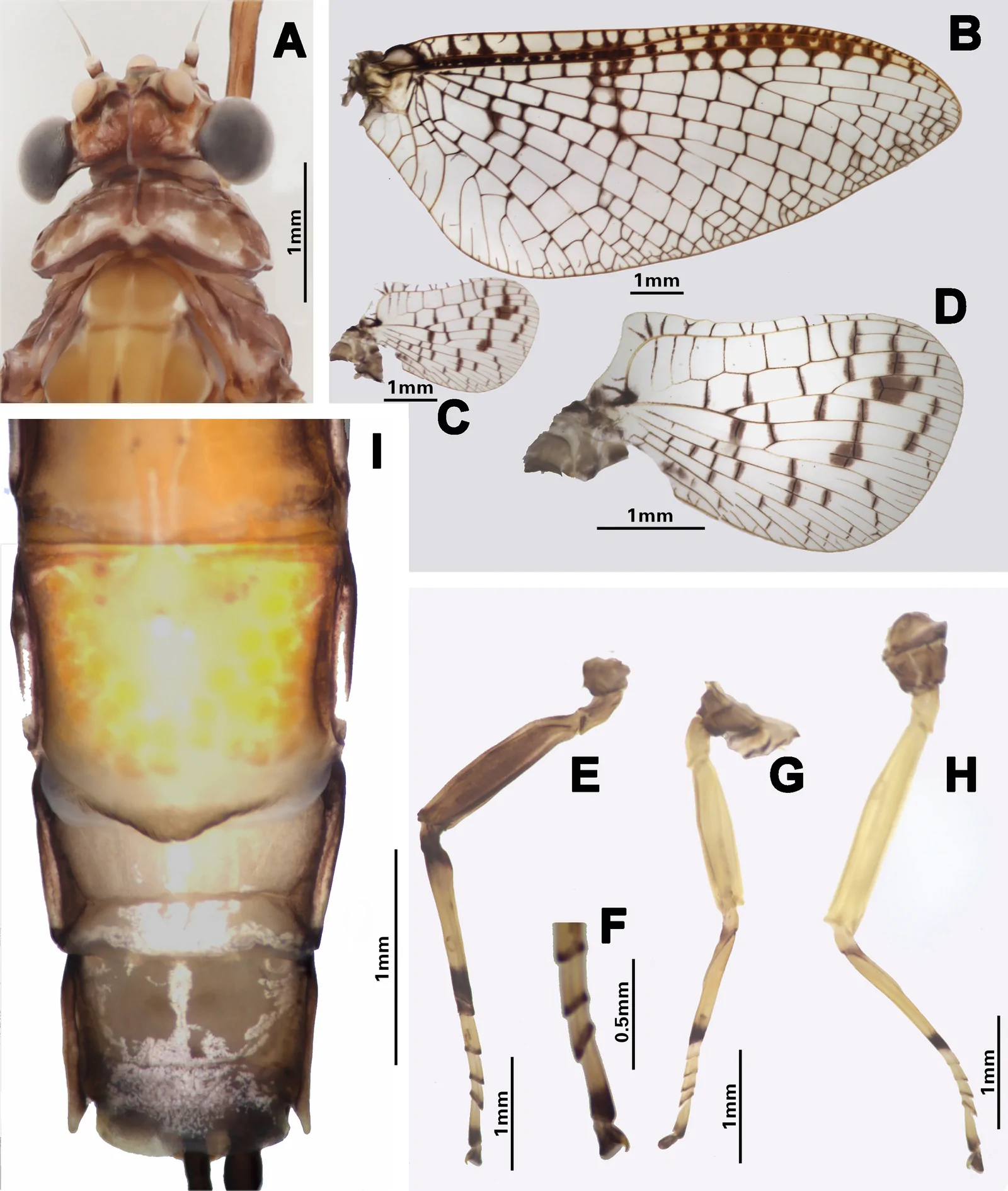
Characters of female imago of *Potamanthellus unicutibius* Nguyen & Bae, 2003 **A** Head (dorsal view); **B** Fore-wing; **C** Hind-wing; **D** Hind-wing enlarged; **E** Fore-leg; **F** Fore-claw; **G** Mid-leg; **H** Hind-leg; **I** Terminal abdoman (ventral view).

Female imago (in alcohol): body length 11.0–12.0 mm, fore-legs 5.0–6.0 mm, fore-wings 11.5–12.0 mm, hind-wings 3.5–4.5 mm, cerci 18.0–21.0, terminal filament 1.8–2.0 mm; body similar to male, but markings of wings slightly paler (Fig. 1B, G, H and Fig. 3B–D). Shortest distance between two compound eyes about 1.8x eye diameter (Fig. 3A). Length ratio of fore-femora: tibiae: tarsi = 1.0: 0.8: 0.8, length ratio of five segments of fore-tarsi = 1.0: 2.5: 2.0: 1.0: 3.5, Fig. 3E–F); length ratio of mid-femora: tibiae: tarsi = 1.0: 0.9: 0.5, length ratio of four movable segments of mid-tarsi =1.0: 1.0: 0.6: 2.5 (Fig. 3G); length ratio of hind-femora: tibiae: tarsi = 1.0: 0.7: 0.4, length ratio of four movable segments of mid-tarsi = 1.0: 1.0: 0.8: 3.0 (Fig. 3H). Two claws of all legs dissimilar, one acute, one blunt (Fig. 3F). Sternum VII slightly thickened and extended posteriorly, forming a triangular lobe (Fig. 3I). Posterior margin of sternum IX slightly extended laterally, median portion slightly concave (Fig. 3I). Terminal filament with 10–12 segments (Fig. 1G and H).

Male subimago (Fig. 1C): body length 9.0 mm, fore-legs 5.0 mm, fore-wings 9.0 mm, hind-wings 3.5 mm, cerci 8.0 mm, terminal filament 0.5 mm. Shortest distance between two compound eyes about 0.67x eye diameter. Length ratio of fore-femora: tibiae: tarsi = 1.0: 1.1: 1.5, length ratio of mid-femora: tibiae: tarsi = 1.0: 0.7: 0.6. Hind-legs similar to mid-legs.

Female subimago (Fig. 1D): body length 11.0 mm, fore-legs 4.0 mm, fore-wings 11.0 mm, hind-wings 3.5 mm, cerci 7.0 mm, terminal filament 1.0 mm. Shortest distance between two compound eyes about 2.0x eye diameter. Length ratio of fore-femora: tibiae: tarsi = 1.0: 1.1: 1.5, length ratio of mid-femora: tibiae: tarsi = 1.0: 0.7: 0.7. Mid- and hind-legs similar to fore-legs, but paler.

Mature nymph (in alcohol): (see Nguyen and Bae (2003) for full description).

**Figure 4.**
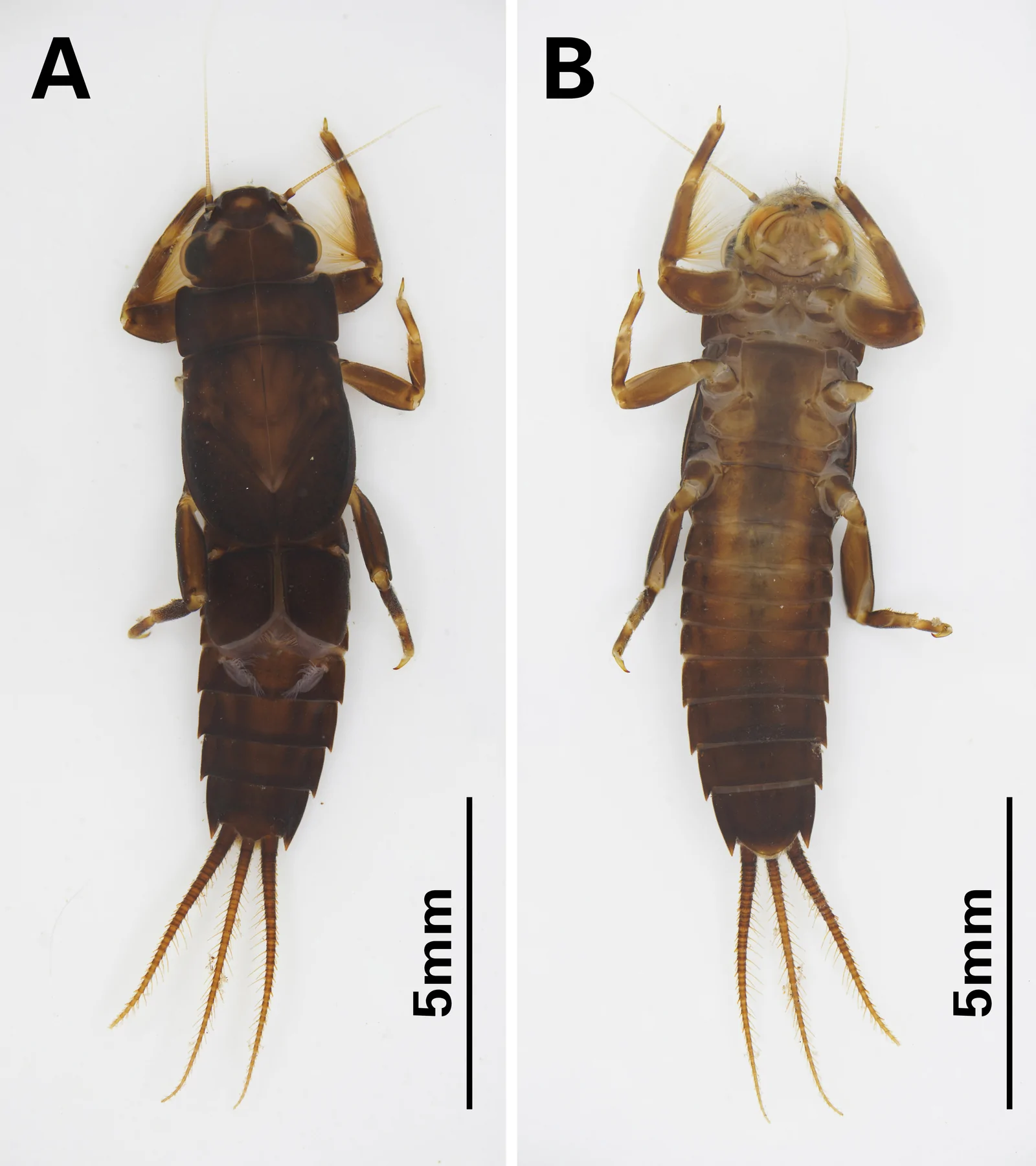
Nymphal habitus of *Potamanthellus unicutibius* Nguyen & Bae, 2003 **A** Dorsal view; **B** Ventral view.

**Figure 5.**
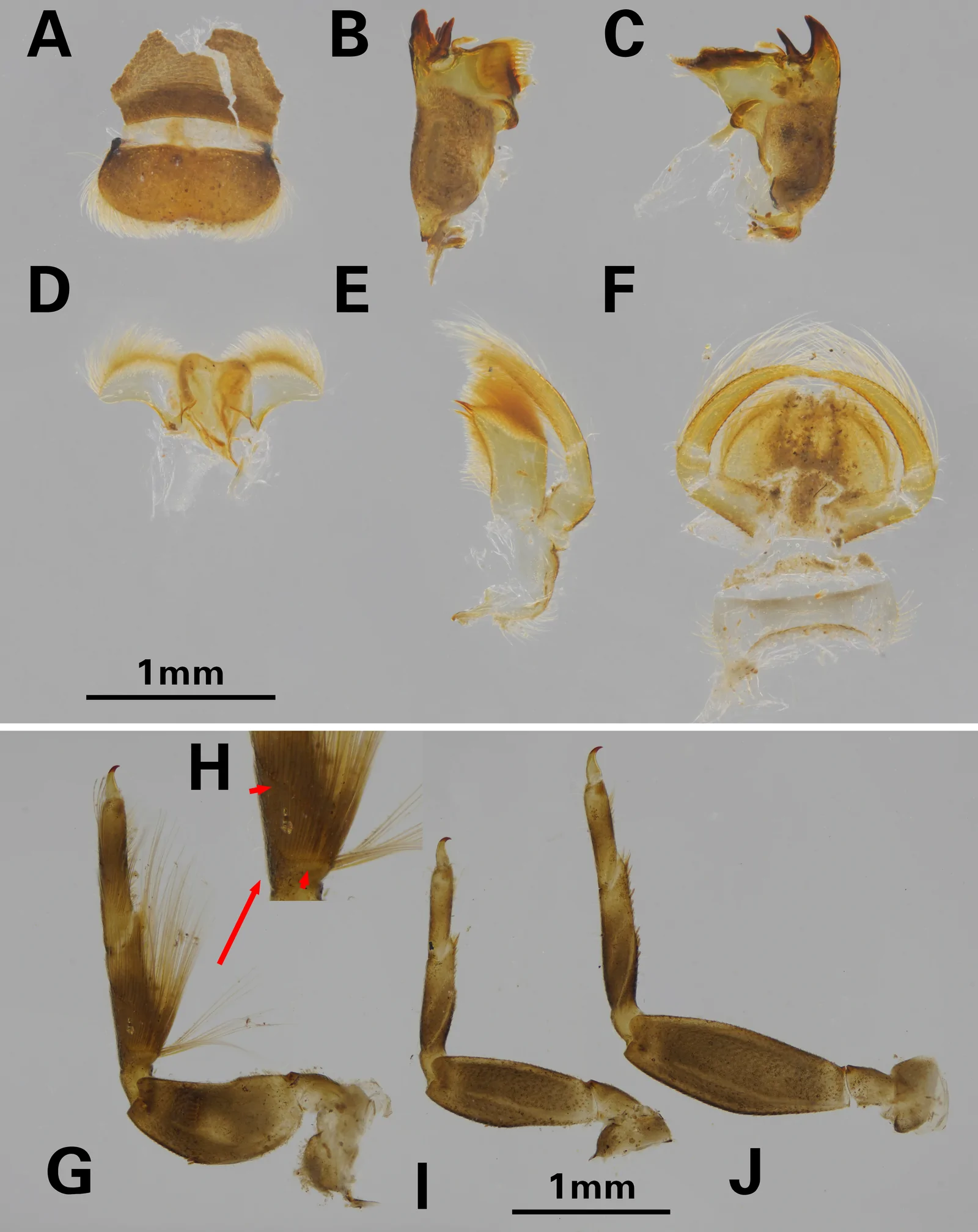
Mouthparts and legs of nymph of *Potamanthellus unicutibius* Nguyen & Bae, 2003 **A** Labrum (dorsal view); **B** Left mandible; **C** Right mandible; **D** Hypopharynx (ventral view); **E** Maxilla; **F** Labium (ventral view); **G** Fore-leg; **H** Fore-tibia enlarged (arrows indicating the setaous rows); **I** Mid-leg; **J** Hind-leg

**Figure 6.**
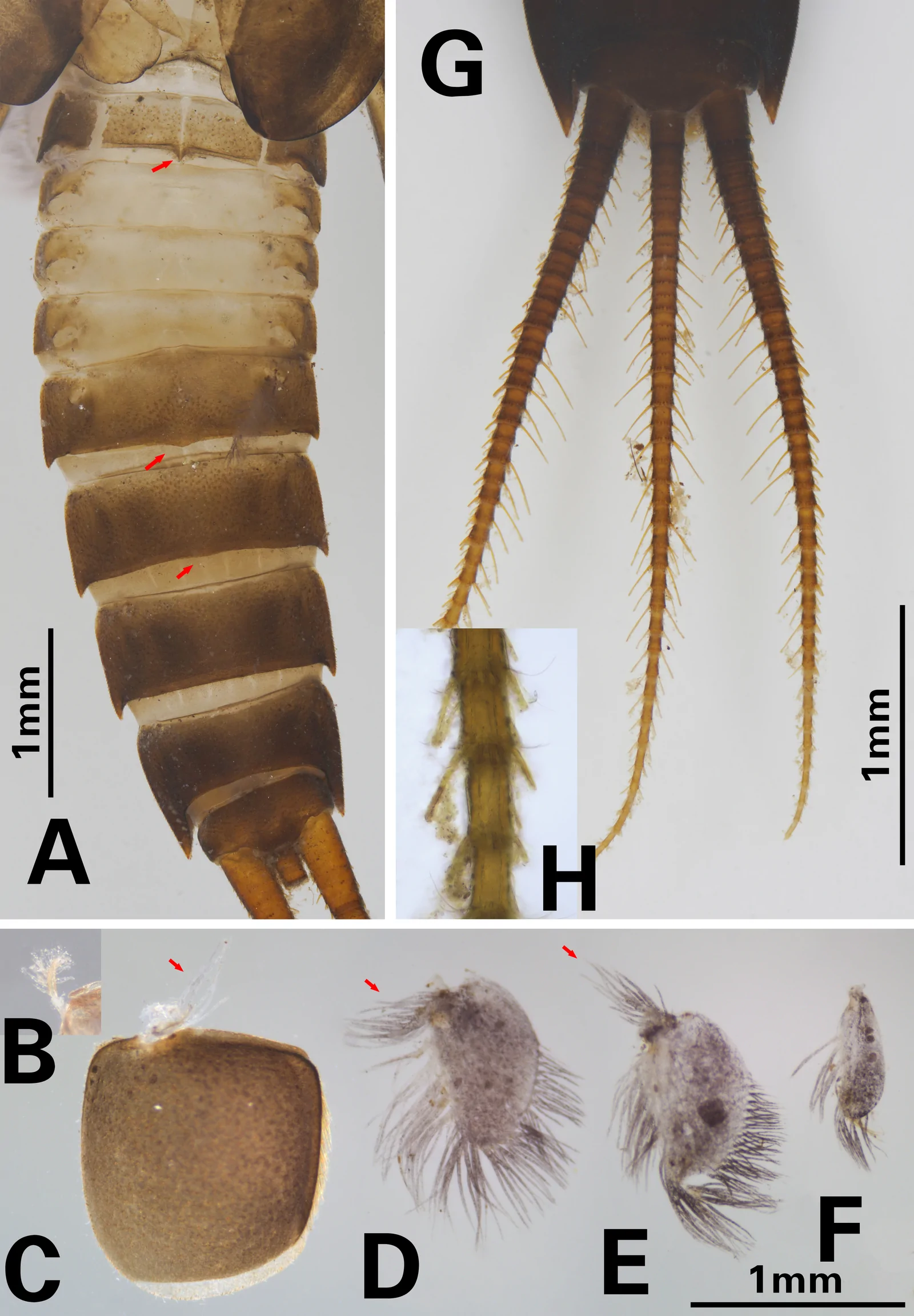
Nymphal characters of *Potamanthellus unicutibius* Nguyen & Bae, 2003 **A** Abdominal terga (arrows indicating the median tubercle); **B** Gills I; **C** Gills II (dorsal view, arrow indicating the ventral lamella); **D** Gills III (arrow indicating the ventral lamella); **E** Gills IV (arrow indicating the ventral lamella); **F** Gills VI; **G** Caudal filaments; **H** Cerci enlarged (showing the spines and setae).

Main characters include: body length 8.0–12.0 mm, caudal filaments 3.0–4.0 mm. Body reddish-brown to chocolate (Fig. 4). Mouthparts as in Fig. 5A–F, filtering mouthparts, maxillary and labial palpi elongated and with dense long hair–like setae, especially their apical segments, length ration of three segments of maxillary palpi = 1.0: 1.5: 4 (Fig. 5E), ratio of labial palpi = 1.0: 0.3: 2.0 (Fig. 5F); fore-legs with a row of subapical transverse spines (Fig. 5G and H); fore-tibiae with two rows of long hair-like setae on dorsal surface, one transverse, one at base, one oblique row near middle (Fig. 5G and H); fore-tarsi with a transverse row of long setae at base and two rows along dorsal and inner margins (Fig. 5G). Length ratio of fore-femora: tibiae: tarsi = 1.0: 0.9: 1.0 (Fig. 5G), ratio of mid-leg = 1.0: 0.6: 0.6 (Fig. 5I), hind-leg = 1.0: 0.6: 0.4 (Fig. 5J), claws hooked. Terga I–II, VI–VII with a median tubercle respectively, that of tergum II more distinct than others (Fig. 6A). Gills shown in Fig. 6B–F, gills I two segmented, with tiny hair–like setae (Fig. 6B); both dorsal and ventral surface of gill opercula smooth, posterior inner margins with dense setae, ventral lamella with fringes (Fig. 6C); gills III–V similar, with dorsal and ventral lamellae (Fig. 6D and E); gills VI single (Fig. 6F). Caudal filaments with stout spines on articulations, cerci with additional short spines on outer side and long spines on inner side; terminal filament with long spines on both lateral sides; articulations with very sparse tiny hair–like setae, especially apical half (Fig. 6G and H).

**Figure 7.**
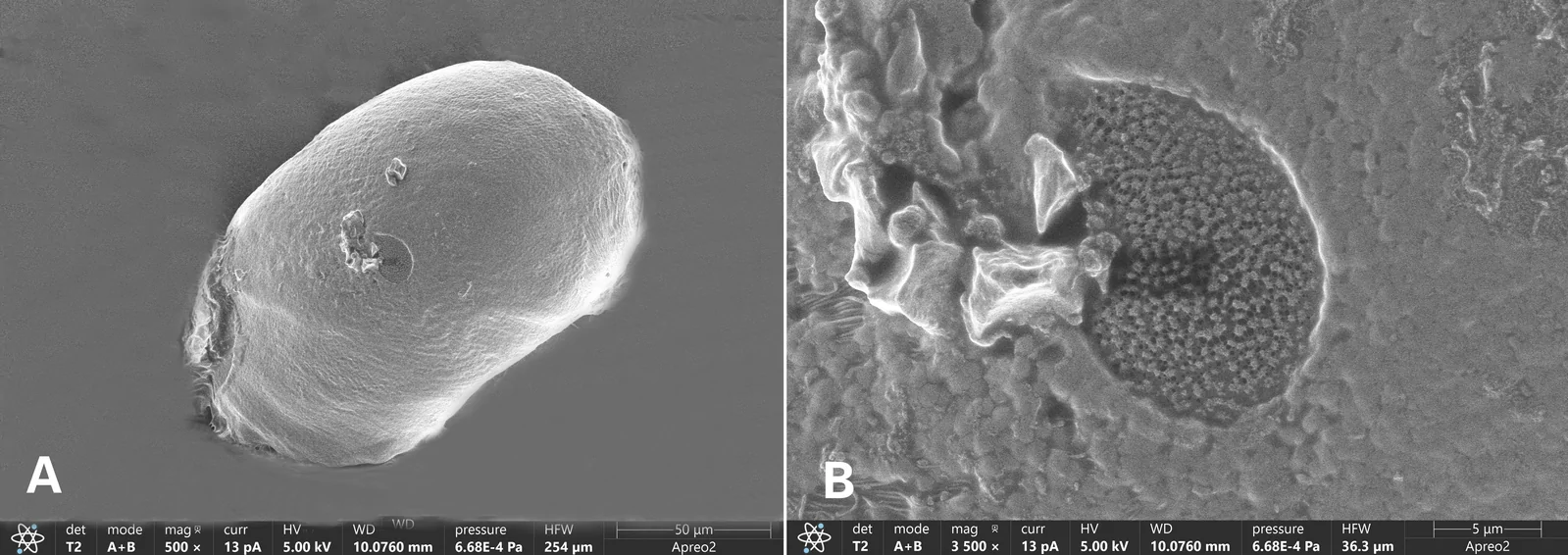
SEM pictures of *Potamanthellus unicutibius* Nguyen & Bae, 2003 **A** Egg; **B** Micophyle.

Egg: surface smooth, micropyle at middle (Fig. 7).

#### Diagnosis

The nymph of *P. unicutibius* can be recognised by its filtering mouthparts and fore-legs: apical segment of maxillary and labial palpi more than 2 times of segment II in length, with dense hairlike setae (Fig. 5E**–**F); fore-tibiae with two oblique rows of long filtering setae (Fig. 5G-H), fore-tarsi with filtering hairs (Fig. 5G). In addition, their relatively short caudal filaments, smooth body and gill opercula, independent gills II, neither pronotum nor mesonotum with distinct expanded anterolateral corner are useful to their identification (Fig. 4).

In imaginal stage, its relatively short body (10.0**–**12.0 mm), heavily stained wings (Fig. 1A**–**B), long fore-legs and its tibiae (longer than 2.0x fore-femora, Fig. 2E) and widely opened compound eyes (Fig. 2A) are good identical characteristics. Its male imago has reduced genitalia, but all components distinct, especially its penes (Fig. 2J**–**L).

**Figure 8.**
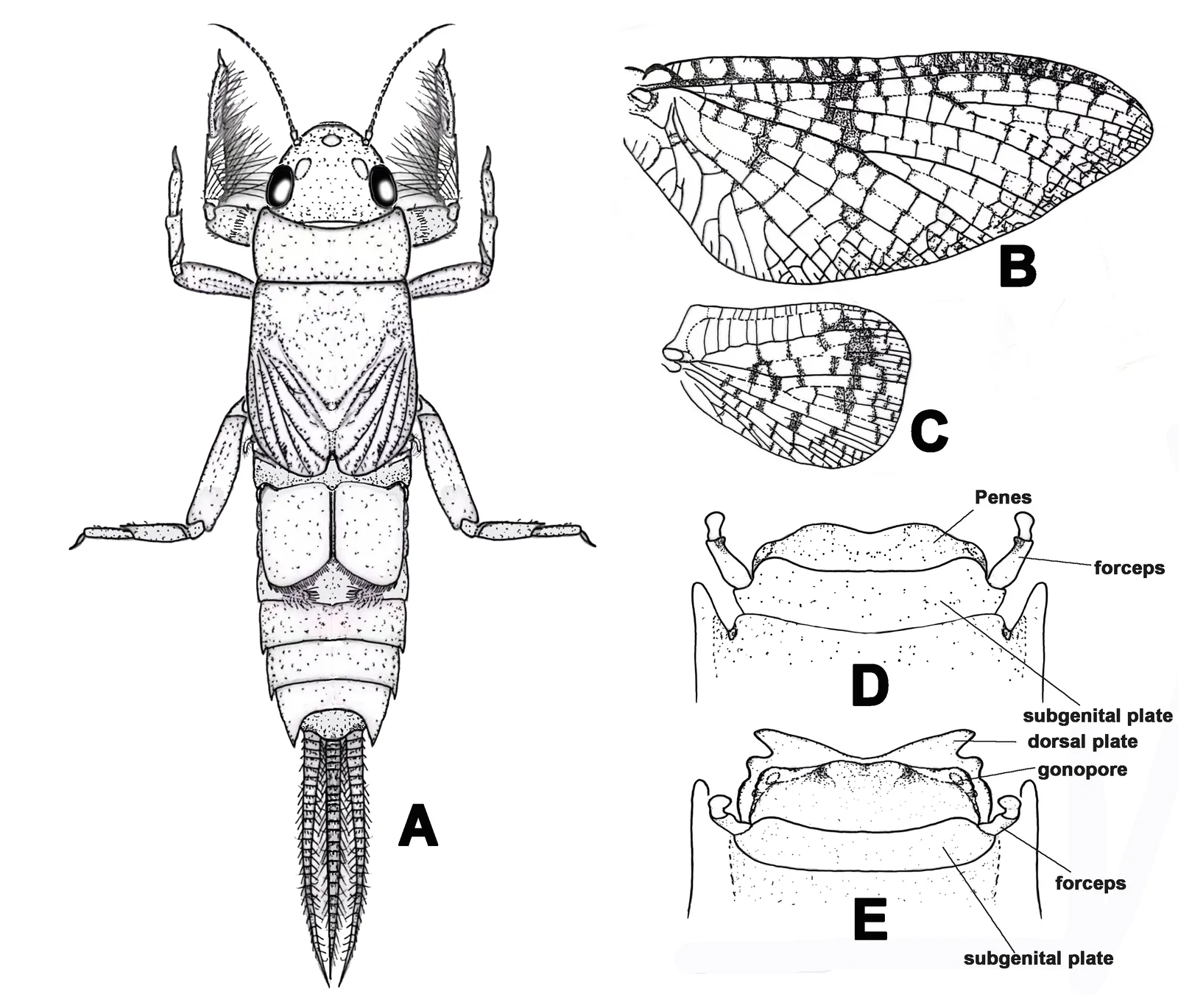
Drawing pictures of *Potamanthellus unicutibius* Nguyen & Bae, 2003 **A** Nymphal habitus (dorsal view); **B** Fore-wing of male imago; **C** Hind-wing of male imago; **D** Male genitalia (ventral view, showing the forceps and subgenital plate); **E** Male genitalia (apicodorsal view, showing the penes and dorsal plate).

All other congeners in the genus *Potamanthellus* have no filtering setae on their fore-leg or mouthparts in nymphal stage. In imaginal stage, all other species have no distinct penes, only forceps and dorsal plate are visible. In addition, the dorsal plates of those species are different: *P. amabilis* and *P. chinensis* have deeply divided plates and their forceps are three-segmented. Although *P. caenoides* has two-segmented forceps, its dorsal plate is two semi-circular (see Bae and McCafferty (1998)). In contrast, P. *unicutibius* has two-segmented forceps and its dorsal plate has two distinct posterolateral processes (Fig. 2L and Fig. 8E).

Egg: surface smooth, without distinct mesh or KCTs (knob-terminated coiled threads), micropyle at equator (Fig. 7).

#### Distribution

Yunnan Prov. (China); Lao Cai Prov. (Vietnam).

#### Ecology

**Figure 9.**
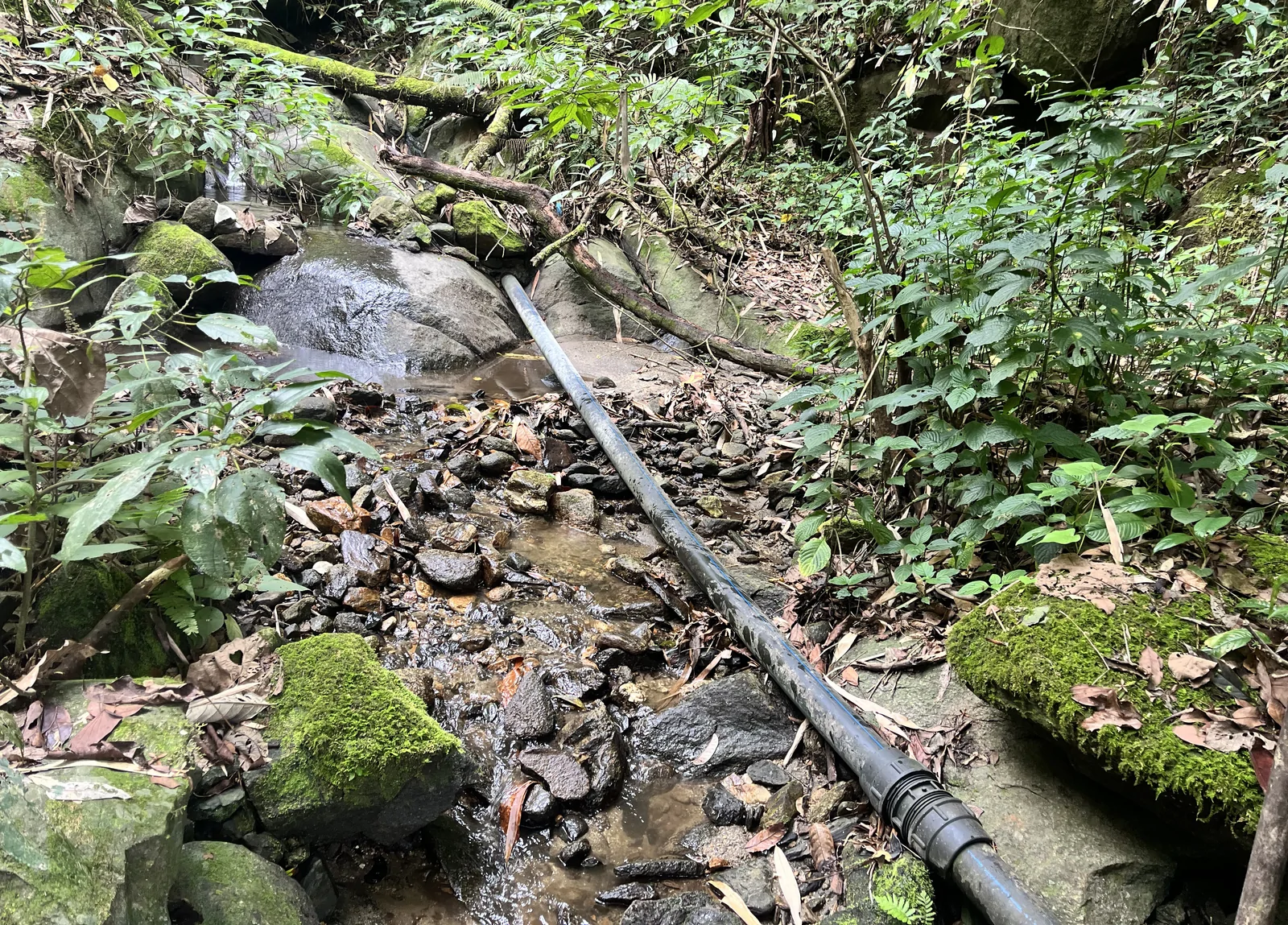
Nymphal Habitat of *Potamanthellus unicutibius* Nguyen & Bae, 2003

This nymphs were found in a narrow creek with large or small stones, mountain area. The width of the creek is about 1–2 m and the flow velocity is 1 m/s (Fig. 9). An *Isonychia* sp. was also found in the same water.

## Discussion

Reduction or vestigialiation of male genitalia, particularly the penes, has been documented in several mayfly lineages. For instance, species within the taxon *Cryptopenella* of *Choroterpes* (Leptophlebiidae) possess indistinct penes, yet retain well-developed forceps and a distinct ventral penis cover (Zhou 2006). Similarly, most species in Baetidae have concealed membranous penes, but their ventral penis cover and forceps remain prominent (Keffermüller 1960, Ying et al. 2021, Wang et al. 2025). In contrast, species of Caenidae typically exhibit minute forceps, while retaining recognisable fused penes (Sun et al. 2026). Surprisingly, *Potamanthellus unicutibius* displays yet another pattern of vestigial genitalia: its forceps, penes and penis cover (a component of the subgenital plate) are all reduced, while it possesses a distinct dorsal covering plate (Fig. 2J–L).

In most mayflies, the gonopores open at the apex of the penes, implying that the penes are three-dimensional structures. However, based on historical accounts (Hsu 1936, Tiunova 1991, Bae and McCafferty 1998) and our own observations of *Potamanthellus* materials, the penes in this genus are membranous and plate-like. In light of the discovery of male *P. unicutibius*, we interpret the structure previously regarded as the penes of *Potamanthellus* to be, in fact, the dorsal plate of the penes, as observed in *P. unicutibius* (Fig. 2J–L).

The genital morphology of *P. unicutibius* may reflect a possible evolutionary trend within the Neoephemeridae: initially, the forceps and penes are relatively well developed, as seen in *Ephemera* or *Potamanthodes* (Lei and Zhou 2024, Qu et al. 2026); later, the penes undergo reduction, as observed in *Pulchephemera* or *Ochernova* (Ma and Zhou 2021, Li et al. 2026); and finally, the forceps become diminished and the penes membranous, as in *P. unicutibius*.

Within the *Neoephemera*-like clade, at least three nymphal morphotypes or evolutionary trends have been recognised: flattened body form (*Pulchephemera*), stout body form (*Leucorhoenanthus* and *Neoephemera*) and slender body form (*Ochernova*) (Li et al. 2026). In contrast, the smooth body surface, operculate gills and swimming-adapted caudal filaments of *Potamanthellus* suggest a certain degree of active locomotion or swimming capability in aquatic environments. Intriguingly, the nymphs of *P. unicutibius* appear to represent a further specialisation, as they are theoretically capable of filtering particulate matter from flowing water. The adult morphology of this species may offer additional evidence bearing on its evolutionary position.

From a biological perspective, based on general nymphal morphology, *P. unicutibius* and other *Potamanthellus* species appear to have evolved in different ecological directions. *P. unicutibius* may be adapted to filtering particulate matter in flowing water, whereas other *Potamanthellus* species likely exhibits greater swimming ability and may feed by chewing on fallen leaves or aquatic macrophytes.

## Supplementary Material

XML Treatment for Potamanthellus unicutibius

## References

[B14406760] Bae Y. J., McCafferty W. P. (1998). Phylogenetic systematics and biogeography of the Neoephemeridae (Ephemeroptera: Pannota). Aquatic Insects.

[B14406769] Chen J. Y., Bian X. N., Zhou C. F. (2025). The exact morphology of *Oligoneuriella
pallida* (Hagen, 1855) from China (Ephemeroptera: Oligoneuriidae). Zootaxa.

[B14406778] Garces J. M., Sartori M. (2022). Neoephemeridae (Insecta, Ephemeroptera) of the Philippines and insights on Southeast Asian biodiversity. Tijdschrift voor Entomologie.

[B14406913] Hsu Y. C. (1936). New Chinese mayflies from Kiangsi province (Ephemeroptera). Peking Natural History Bulletin.

[B14406922] Keffermüller M. (1960). Badnania nad fauna jętek (Ephemeroptera) Wielkopolski. Prace Komisji Biologicznej Pozna skie Towarzystwo Przyjaciól-Nauk.. Wydzial-Matematyczno Przyrodniczy.

[B14406787] Kluge N. J. (2004). The phylogenetic system of Ephemeroptera.

[B14406813] Lei Z. M., Zhou C. F. (2024). The subgenus *Sinephemera* Kluge, 2004 in China (Ephemeroptera: Ephemeridae: Ephemera Linnaeus, 1758). Zootaxa.

[B14406949] Lestage J. A. (1931). Contribution à l'étude des larves des Éphéméroptères. VII. Le groupe Potamanthidien. Mémories de la Société entomologique de Belgique.

[B14406822] Li M. Y., Deng M. H., Zhou C. F. (2026). The newly found imagoes of *Ochernova
tshernovae* (Kazlauskas, 1963) from China validating its position in the family (Ephemeroptera: Neoephemeridae). Zootaxa.

[B14406831] Ma Z. X., Zhou C. F. (2021). The imaginal characters of *Neoephemera
projecta* showing its plesiomorphic position and a new genus status in the family (Ephemeroptera: Neoephemeridae). Insects.

[B14406849] Mcdunnough J. (1925). New Canadian Ephemeridae with notes, III. The Canadian Entomologist.

[B14406931] Nguyen V. V., Bae Y. J. (2003). Taxonomic review of the Vietnamese Neoephemeridae (Ephemeroptera) with description of *Potamanthellus
unicutibius*, new species.. Pan-Pacific Entomol.

[B14406858] Qiang X. H., Zhou C. F. (2023). A preliminary review of *Isonychia* Eaton, 1871 from Chinese mainland with a re-description of *I.
kiangsinensis* Hsu, 1936 (Insecta, Ephemeroptera, Isonychiidae). ZooKeys.

[B14406867] Qu J. L., Zhu Y. N., Zhou C. F. (2026). A new *Potamanthodes* Ulmer, 1920 species from central China (Ephemeroptera: Potamanthidae). Journal of Insect Biodiversity.

[B14406876] Sun Y. X., Deng M. H., Zhou C. F. (2026). First imago descriptions for *Brachoculis
acutalis* (Zhou, Sun, and McCafferty, 2003) with potential implications for the higher classification of Caenidae (Ephemeroptera). Aquatic Insects.

[B14406940] Tiunova T. M. (1991). Description of imago *Potamanthellus
rarus* (Ephemeroptera, Neoephemeridae) from Primorye.. Zoologicheskii Zhurnal (Moscow).

[B14406885] Wang N. N, Wang D., Gattolliat Jean-Luc, Suttinun Chanaporn, Zhou C. F. (2025). First record of the adult of *Cymbalcloeon* confirming its position in Baetidae (Insecta, Ephemeroptera). Biodiversity Data Journal.

[B14406895] Ying X. L., Li W. J., Zhou C. F. (2021). A review of the genus *Cloeon* from Chinese mainland (Ephemeroptera: Baetidae). Insects.

[B14406904] Zhou C. F. (2006). The status of *Cryptopenella* Gillies, with description of a new species from Southwestern China (Ephemeroptera: Leptophlebiidae). Oriental Insects.

